# Managing the risk of suicide in a psychiatry clinic: an ethnographic study on the work atmosphere of nurses during the pandemic period

**DOI:** 10.1192/j.eurpsy.2023.1062

**Published:** 2023-07-19

**Authors:** S. Öztürk, D. Hİçdurmaz, M. Soileau

**Affiliations:** Psychiatric Nursing, Hacettepe University Nursing Faculty; 2 Anthropologist, Independent Scholar, Ankara, Türkiye

## Abstract

**Introduction:**

It is known that the restrictions and clinical changes experienced during the pandemic period have negative effects on the care and treatment of psychiatric patients. However, insights on how the pandemic environment and the approaches of healthcare professionals serving during the pandemic affect the care and treatment of patients at risk of suicide are insufficient.

**Objectives:**

This ethnographic study aimed to identify the approaches of psychiatric nurses in managing suicide risk during the pandemic period in relation with their work environment.

**Methods:**

This ethnographic research design used a sample of 13 psychiatric nurses in a psychiatric clinic in Ankara. Data were collected with in-depth interviews, participant observations and observant participations. Data were obtained from a total of 612 hours of observation and 13 planned nurse interviews. Data were analyzed using qualitative thematic analysis.

**Results:**

The emergent main theme from data analysis is explained under the title of “The risk of loss of inauthenticity in suicide risk management”. “Risk of loss of inauthenticity” means the fact that the individual suicidal risk factors and differences of the patients cannot be perceived/assessed by the nurses.

**Conclusions:**

Supposing all patients having the same suicide risk level by psychiatric nurses caused insensitivity to risky patients in the care process. Nurses’ inadequate approach to patient personality disorder and limited social interactions due to the pandemic atmosphere made it difficult for nurses to have knowledge and understanding of how patients cope with suicidal ideations. These findings show the importance of the use of valid and reliable scales with risk formulations and the significance of triage in crisis periods such as current pandemic. In addition, creating available online consultancy service alternatives may have an important role in the management of suicide risk for patients who are disturbed by long-term hospitalization. Also these findings may contribute to the creation of qualified care and treatment guidelines on suicide risk management for crisis periods.

**Disclosure of Interest:**

None Declared

